# Earl Grey: A Fully Automated User-Friendly Transposable Element Annotation and Analysis Pipeline

**DOI:** 10.1093/molbev/msae068

**Published:** 2024-04-05

**Authors:** Tobias Baril, James Galbraith, Alex Hayward

**Affiliations:** Centre for Ecology and Conservation, University of Exeter, Penryn Campus, Cornwall TR10 9FE, UK; Laboratory of Evolutionary Genetics, Institute of Biology, University of Neuchâtel, 2000 Neuchâtel, Switzerland; Centre for Ecology and Conservation, University of Exeter, Penryn Campus, Cornwall TR10 9FE, UK; Institute of Evolutionary Biology, University of Edinburgh, Edinburgh EH9 3FL, UK; Centre for Ecology and Conservation, University of Exeter, Penryn Campus, Cornwall TR10 9FE, UK

**Keywords:** transposon, repeat, mobile genetic element, genome, software, consensus

## Abstract

Transposable elements (TEs) are major components of eukaryotic genomes and are implicated in a range of evolutionary processes. Yet, TE annotation and characterization remain challenging, particularly for nonspecialists, since existing pipelines are typically complicated to install, run, and extract data from. Current methods of automated TE annotation are also subject to issues that reduce overall quality, particularly (i) fragmented and overlapping TE annotations, leading to erroneous estimates of TE count and coverage, and (ii) repeat models represented by short sections of total TE length, with poor capture of 5′ and 3′ ends. To address these issues, we present Earl Grey, a fully automated TE annotation pipeline designed for user-friendly curation and annotation of TEs in eukaryotic genome assemblies. Using nine simulated genomes and an annotation of *Drosophila melanogaster*, we show that Earl Grey outperforms current widely used TE annotation methodologies in ameliorating the issues mentioned above while scoring highly in benchmarking for TE annotation and classification and being robust across genomic contexts. Earl Grey provides a comprehensive and fully automated TE annotation toolkit that provides researchers with paper-ready summary figures and outputs in standard formats compatible with other bioinformatics tools. Earl Grey has a modular format, with great scope for the inclusion of additional modules focused on further quality control and tailored analyses in future releases.

## Introduction

Over recent decades, great advances in genome sequencing technologies have accompanied significant decreases in sequencing costs. This has led to a huge increase in the availability of genome assemblies for lineages across the eukaryotic tree of life. Further, the advent of long-read sequencing technologies has resulted in a wealth of high-quality and highly contiguous genome assemblies, with chromosome-level resources becoming increasingly common for both model and nonmodel organisms. The massive availability of high-quality genomic resources for diverse organisms provides an exciting opportunity to further our understanding of eukaryote evolution and in turn the evolution and diversity of the transposable elements (TEs) hosted within eukaryotic genomes. TEs are DNA sequences that are capable of moving from one location to another in a host genome, with their own distinct evolutionary trajectories (McClintock 1956). It is clear that TEs are major components of genome content across the eukaryotic tree of life, and that TEs are a major reservoir of genetic variation (Wells and Feschotte 2020). As such, TEs are implicated in a range of evolutionary processes involved in the generation of genomic novelty via processes including alteration of coding sequence, chromosomal rearrangements, and modification of gene regulatory networks (Chung et al. 2007; Van’t Hof et al. 2016; Chuong et al. 2017; Cosby et al. 2019). The number of host processes in which TEs are implicated continues to grow at a high pace, and the extent to which TEs play a fundamental role in eukaryotic evolution remains an open question.

The complex repetitive nature of TEs previously made their assembly from short reads challenging, as repetitive regions were often collapsed or unresolved in genome assemblies. However, the increasing availability of long-read genomic resources provides a timely opportunity to significantly improve our understanding of TE evolution and diversity through studies that sample genomes at varying scales, from population-wide to kingdom-wide and beyond. However, to do this efficiently and cope with the flood of sequencing data currently being generated, high-throughput automated methodologies are essential.

Tools for annotating the location and identity of TEs within a genome are a basic aspect of genomics, even where there is little ultimate research focus on TE biology. A key example is the assembly of a new genome, where TE annotation is performed so that TEs do not interfere with gene prediction pipelines (Campbell et al. 2014; Kollmar 2019). This involves screening for TEs present in the genome and masking them, so they are filtered out during subsequent steps, typically using annotations that replace their nucleotide sequences with either lowercase letters (softmasking) or another symbol such as “N” or “X” (hardmasking). Given that TE annotation is typically carried out prior to gene prediction, the quality of TE annotation performed can influence the resultant quality of host gene annotation. For example, many TEs contain protein-coding regions that could be incorrectly annotated as host genes (Bourque et al. 2018), while repetitive host gene families can be mistaken for TEs.

TEs are inherently repetitive in nature, and many TE annotation approaches firstly seek to identify “families” of elements composed of closely related TE copies considered to originate from a single initial integration in the genome of interest. TE families are typically defined by thresholds, such that two TE sequences are considered to belong to the same family if they align with at least 80% identity, across at least 80 bp, and at least 80% of their length (Wicker et al. 2007). Resultant TE families are commonly represented by a single consensus sequence, constructed by aligning multiple copies sampled from the genome that fall within the defined sequence threshold. Collectively, the set of resultant TE models for a given species is referred to as a “TE library.” TE families can be identified using “library-based” approaches, which search the genome for similarity to elements already present in TE databases. Alternatively, “de novo” analyses use either existing knowledge of TE structure or their repetitive nature to detect TEs within a genome.

One of the most well-known and frequently employed automated TE annotation workflows involves de novo TE family curation with RepeatModeler (and more recently RepeatModeler2; Flynn et al. 2020), followed by annotation of the host genome with the newly created de novo library using RepeatMasker (Smit et al. 2013). This approach provides an overview of repeat content, but leaves considerable room for improvement in several areas, including the quality and completeness of TE consensus generation, TE classification, and subsequent host genome TE annotation. The gold standard for TE annotation is manual curation, whereby a de novo TE prediction program is run on a genome assembly to identify putative TE sequences, before individual TE copies are extracted and a multiple alignment is generated for each TE “family” (Goubert et al. 2022). These alignments are then curated by eye by a human investigator, which involves identifying hallmarks of TE boundaries (such as target site duplications and long terminal repeats [LTRs]), extending TE alignments as necessary, or trimming alignments to remove poorly aligned regions unlikely to represent true TE sequence. The strength of manual curation lies in the individual care taken to annotate each TE model. However, the inherent difficulties associated with manual curation methods are not easily surmountable. Manual curation requires expert knowledge of the structure and biology of diverse TE types. It also requires significant time investment and is not a feasible option for general application given the rate that new genomic data are being produced. Consequently, manual curation is generally only applicable to studies focusing on single genomes or small sets of genomes. Furthermore, the individual-based nature of manual curation also leads to reproducibility issues due to variability introduced when trimming sequence alignments by eye, especially when considering differences in experience, pattern recognition ability, and human error.

To address the limitations of manual TE curation and facilitate large-scale comparative and population-level studies, a diversity of automated TE annotation tools have been developed (Goerner-Potvin and Bourque 2018). However, many of these tools are species or element specific and need a level of technological expertise to install and configure, often requiring multiple dependencies and databases, which can be difficult to locate and apply due to dead weblinks, a lack of updates, and retirement of codebases/dependencies. Compounding these problems is a lack of focus on user-friendliness in the usage processes, which frequently includes issues relating to data compatibility with downstream tools, necessitating rounds of data reformatting or cleaning. Overall, the above issues can act as a significant barrier to discourage or prohibit interested biologists from performing research on TEs.

Automated TE annotation remains a computationally challenging process. This is due to the great diversity and complexity of TE sequences, the huge number of TEs that likely remain uncharacterized, and the gradual postintegration erosion of TE sequences due to host mutational processes, which reduces the ability to recognize and identify them. Consequently, automated TE annotation tools often struggle to accurately identify TE sequences. Methodologies that generate de novo TE consensus sequences often produce TE consensus libraries with high levels of redundancy, where a single TE is represented by multiple TE consensus models, and these models often perform poorly in their designation of TE boundaries (Rodriguez and Makałowski 2022). Following consensus generation, TE annotation can also lead to challenges that affect both de novo and library-based methods. Fragmented TE annotations are often inferred, where a single TE is represented by multiple separate annotations instead of a single continuous annotation. In addition, TE annotations often contain overlaps, where a single base pair is annotated with multiple TE identities. Such issues can result in distorted TE counts and coverage estimates, with knock-on implications for downstream analyses. Many of these issues are particularly acute when considering nonmodel organisms that are distantly related to model reference species, where considerable effort has been expended to provide high-quality TE curation.

The release of new genome assemblies has outpaced detailed efforts to characterize their associated TEs. Furthermore, this situation will accelerate with the progress of massive-scale genome sequencing efforts such as the Darwin Tree of Life project and the Earth Biogenome Project (Lewin et al. 2018), which seek to provide high-quality chromosomal-level genome assemblies for all eukaryotic life in the British Isles and planet Earth, respectively. Such projects offer huge opportunities to expand our understanding of TE biology. However, the scale of genomic resources available also greatly increases the need for rapid, robust, repeatable, and user-friendly automated TE annotation and analysis approaches, so that genomes are accurately annotated for TEs, and public TE databases are not overwhelmed with poor-quality sequences uploaded from substandard annotations.

Here, we present Earl Grey, a fully automated TE annotation pipeline combining widely used library-based and de novo TE annotation tools, with TE consensus and annotation refinements, aimed at generating high-quality TE libraries, annotations, and analyses for eukaryotic genome assemblies.

## Implementation

To make Earl Grey as user-friendly as possible, we have provided several solutions to suit the needs of most researchers. Earl Grey is available from a Github repository (https://github.com/TobyBaril/EarlGrey), as a package in Bioconda (Grüning et al. 2018) (recommended installation), as a preconfigured Docker/Singularity container (https://biocontainers.pro/tools/earlgrey), and in a web browser via gitpod (https://gitpod.io). It can be run on Linux distributions, such as Ubuntu, and installed on a local system or HPCs supporting Conda, Docker, or Singularity. Earl Grey is parallelized and makes use of multiple CPU threads to reduce runtime.

Earl Grey runs in a configured environment to avoid conflicts between tool versions and to streamline the installation procedure. Users who do not have RepeatMasker (Smit et al. 2013) and RepeatModeler2 (Flynn et al. 2020) installed, and who do not wish to do this, can install Earl Grey using the Bioconda package or via interactive containers with Docker and Singularity, which will install and configure dependencies automatically and provide a virtual machine in which to perform all Earl Grey analyses. For installation directly from GitHub, it is necessary to have RepeatMasker and RepeatModeler2 preinstalled. Instructions for the installation of dependencies are provided for users who do not currently have them installed. In an effort to maintain Earl Grey as open source, Earl Grey has been tested and is recommended to be used, with the curated subset of the Dfam database of repetitive DNA elements (Hubley et al. 2016; tested with all versions from release 3.6 onwards: earlier versions are not compatible due to the transition of RepeatMasker libraries to the H5 format). If users have access to RepBase RepeatMasker edition libraries (Jurka et al. 2005; Kapitonov and Jurka 2008), they can also choose to configure RepeatMasker with these in addition to Dfam.

Once installed and configured, Earl Grey will run on a given input genome assembly in FASTA format with a single command. Prior to running Earl Grey, the “earlgrey” conda environment must be activated “conda activate earlgrey.” Once the conda environment is active, Earl Grey can be called with the command “earlGrey.” There are three required options and six optional parameters (Table 1). For example, a run on the *Homo sapiens* genome, with the genome located in the current directory, could be started with the minimum parameters: “earlGrey -g homoSapiens.fasta -s homoSapiens -o ./homoSapiens_outputs/.” If users require the curation of a TE library for a polyploid species, we recommend providing Earl Grey with a haploid genome assembly for TE library construction, followed by annotation of the full polyploid assembly.

**Table 1:** Parameters for Earl Grey

Command flag	Description	Required?
-g	Path to genome FASTA file.	Y
-s	Name for results files (cannot contain spaces).	Y
-o	Path to output directory (. represents current directory). Relative file paths can be used.	Y
-r	RepeatMasker species search term used for the optional initial mask of known elements, in string (“Arthropoda”) or NCBI taxonomy ID (6656) format.	N
-l	User-provided custom TE library in FASTA format for the optional initial mask of known elements.	N
-t	Number of threads (default: 1).	N
-c	Should the de novo consensus sequences be clustered to reduced library redundancy? (yes|no, default: no).	N
-m	Do you want to remove all potentially spurious TE annotations <100bp? (yes|no, default: no).	N
-d	Generate a softmasked genome after annotation? (yes|no, default: no).	N
-i	Number of iterations to run the BLAST, Extract, Align, Trim process (default: 10).	N
-f	Number of flanking bases to add in each round of the BLAST, Extract, Align, Trim process (default: 1000).	N
-n	Maximum number of sequences to use during consensus improvement during the BLAST, Extract, Align, Trim process (default: 20).	N

**Fig. 1.**
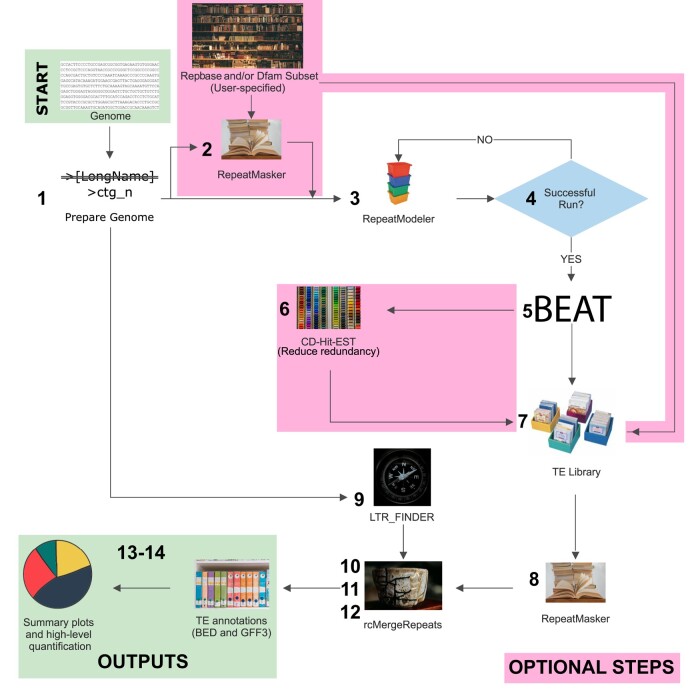
Flowchart illustrating the Earl Grey workflow. Processing is fully automated from start to finish. Stage numbers correspond to steps in the main text.

**Fig. 2.**
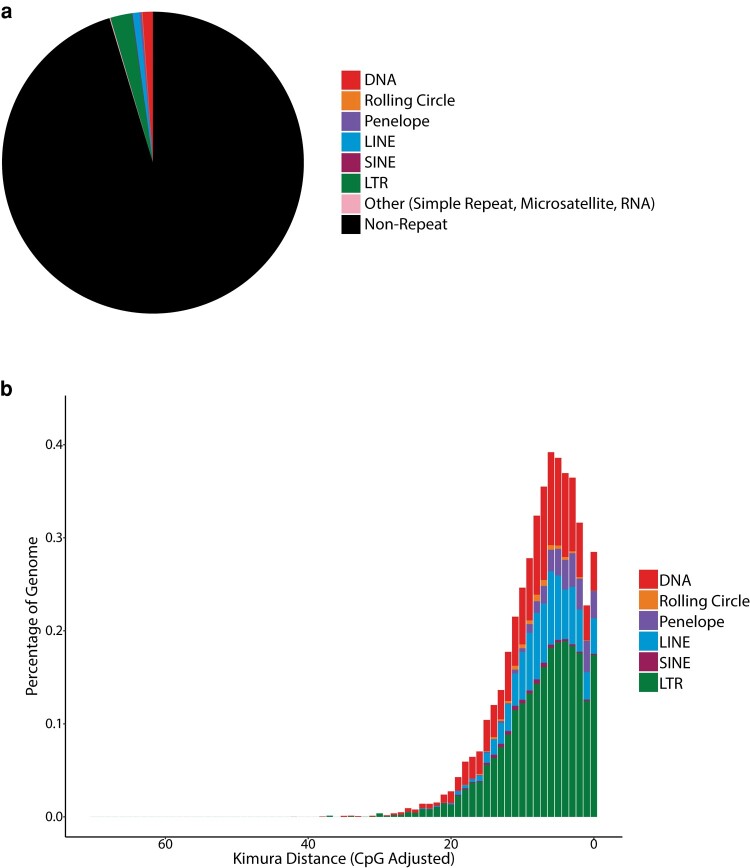
Example summary figures produced by Earl Grey using the results from the annotation of an 840 Mb simulated genome with 58% GC content. a) Summary pie chart showing the proportion of the genome annotated with the main TE classifications. b) Repeat landscape plot, where the *x* axis indicates divergence from consensus in Kimura distance, and the *y* axis indicates the percentage of the genome annotated as TE for each level of divergence. The *x* axis is reversed relative to the plots produced when using RepeatMasker, such that ancient activity (greater divergence to consensus) appears on the left-hand side, while more recent activity is shown toward the right (greater similarity to consensus). Landscape plots can be used to provide an estimate of TE activity within a given genome assembly, with recent activity indicated by a low distance from consensus.

Earl Grey runs through a multistep TE curation and annotation pipeline to annotate a given genome assembly with all intermediate results saved in their respective directories (Fig. 1). Logs are printed to “stdout” (the console) and saved to a log file in the Earl Grey output directory. The steps involved in the Earl Grey TE annotation procedure are outlined below:

1. The first step of Earl Grey prepares the input genome for analysis. Some tools used in the pipeline are sensitive to long header names. To prevent associated issues, header names are stored in a dictionary and replaced with generic headers using the naming convention “ctg_n,” where “n” is a unique integer for each entry in the FASTA file. Ambiguous nucleotide IUPAC codes are replaced with “N” due to incompatibility with some tools, including the search engines used by RepeatMasker. The original input genome file is backed up and compressed, with the prepared input genome version saved under the same file name appended with the extension “.prep.”
2. If required by the user, known repeats are identified and masked using RepeatMasker and a user-specified subset of the TE consensus libraries (i.e. Dfam and/or RepBase depending on RepeatMasker configuration) or a user-supplied custom FASTA file of consensus sequences. This is a nondefault option, as in most cases, it is expected that annotation is being performed on a previously unscreened species, and a new search is required, rather than grouping TE families with previously described elements. A sensitive search is performed ignoring small RNA genes “-s -norna,” and a hard masked version of the input genome is produced with nucleotides within known TEs replaced with “N.”
3. The input genome, or masked genome if step 2 is performed, is analyzed with RepeatModeler2 for de novo TE identification (Flynn et al. 2020). The optional LTR identification step included as part of RepeatModeler2 is not used, as we implement a separate LTR curation step later in the Earl Grey pipeline during the RepeatCraft stage, as this is a requirement for RepeatCraft (see step 12). RepeatModeler2 outputs a library of de novo consensus sequences using the following naming convention: “rnd-n_family-n#TE_Classification” (e.g. rnd-1_family-256#LINE/R2-Hero), where rnd stands for the RepeatModeler analysis round in which an element was identified. (The default of six rounds are run in Earl Grey. If annotating very large genomes, manually increasing RepeatModeler2 sampling rounds is recommended.)
4. The success of the RepeatModeler2 run is verified as failures can occur when annotating certain genome assemblies. For example, when annotating a genome where enough unsampled nucleotides remain to initiate a new round of RepeatModeler2, but where there are not enough unsampled long sequences for the additional round to run successfully, this leads to a program failure (e.g. https://github.com/Dfam-consortium/RepeatModeler/issues/118). If this occurs, Earl Grey will automatically restart the RepeatModeler2 run with a reduced maximum stage number to ensure it runs successfully.
5. To generate maximum-length de novo TE consensus sequences, the de novo TE library undergoes an automated implementation of the “BLAST, Extract, Extend” (BEE) process described by Platt et al. (2016). In Earl Grey, this process is termed “BLAST, Extract, Align, Trim” (BEAT) and is adapted from TEstrainer (https://github.com/jamesdgalbraith/TEstrainer). BEAT is iteratively performed on each consensus sequence until either the sequence cannot be considerably improved (based on increases in sequence length) or, to prevent infinite loops, the maximum number of specified iterations is complete. The default parameters used throughout BEAT (including extending flanks by 1,000 bp, a maximum of 10 iterations, and the use of 20 sequences for consensus construction) were selected through extensive manual testing (discussed in supplementary additional file S1). However, all parameters can be specified by the user with command line options. Using default options, the BEAT process proceeds as follows: (i) Each iteration begins with a search for tandem repeats within the consensus sequence using Tandem Repeat Finder (TRF; Benson 1999). If TRF identifies >90% of the consensus sequence as tandem repeats, the consensus is classified as a tandem repeat and not curated further. If 50% to 90% of the consensus sequence is identified as tandem repeats, the longest section not identified as tandem repeats is extracted and treated as the final consensus sequence, and the sequence is not curated further. If TRF finds <50% of the consensus sequence to be tandem repeats, curation proceeds. (ii) Genome-wide copies of the repeat are identified using BLAST+ (Camacho et al. 2009; -task dc-megablast). The top 20 matching sequences (defined as having >70% pairwise identity and >50% coverage of the query consensus sequence) are selected, and both flanks of each of the 20 sequences are extended by 1,000 bp. If at any point of the iterative process filtering reduces the number of genome-wide matches to below three, the iteration's starting consensus sequence is considered as high quality as possible, and further iterative curation ceases. To ensure the selected sequences improve on the original RepeatModeler sequence, the extended sequences are aligned to the initial RepeatModeler consensus sequence using BLAST (-task dc-megablast), and any that do not align with >70% pairwise identity and >50% coverage are discarded. Manual testing found that, without this step, it is possible for the final consensus sequence to occasionally represent a more abundant repetitive sequence that neighbors a copy of the initial RepeatModeler consensus sequence. (iii) The extended sequences are pairwise aligned to each other using BLAST (-task dc-megablast). Using these alignments, the flanking regions of the extended sequences are trimmed to only include sequence that aligns to at least one other extended sequence. A multiple sequence alignment (MSA) of the trimmed, extended sequences and the starting consensus sequence is constructed using MAFFT (Katoh and Standley 2013). Any columns of the resultant MSA that contain only a single nucleotide are removed from the MSA, followed by removal of the starting consensus sequence, so that only the new extended and trimmed sequences remain. This first MSA is realigned with MAFFT, and a new consensus sequence is constructed using majority rule. Manual testing identified fewer ambiguous positions in consensus sequences constructed from realigned MSAs compared to consensus sequences constructed from the first MSA. This new consensus sequence is aligned to the previous iteration's consensus sequence using BLAST (-task dc-megablast) to verify if the sequence is improved. Any new sequence either shorter than or having less than 80% coverage of the previous consensus sequence is treated as a reduction in quality, and the previous iteration's consensus sequence is treated as the final consensus sequence. New consensus sequences longer than the starting sequence by <50% of the flank extension size are treated as complete and not curated further. New consensus sequences longer than the previous consensus sequence by >50% of the flank extension size are submitted for a further round of curation. (iv) Following the iterative BEAT curation process, TRF (Benson 1999), MREPS (Kolpakov et al. 2003), and SA-SSR (Pickett et al. 2016) are used to identify potential satellite repeats in the BEAT curated consensus sequence library. Any consensus sequences identified as >50% tandem repeats are considered likely to be simple or satellite repeats. Within this set, any sequences identified as being >90% a contiguous tandem repeat with a period of 200 bp or more are classified as macrosatellites, and the consensus is trimmed to a single period of the repeat.
6. If clustering is specified by the user, the set of de novo consensus sequences are clustered using cd-hit-est (Li and Godzik 2006; Fu et al. 2012) with parameters satisfying the TE family definition of Wicker et al. (2007), implemented as described by Goubert et al. (2022), i.e. “-d 0 -aS 0.8 -c 0.8 -G 0 -g 1 -b 500 -r 1.’ The motivation behind clustering is to reduce the presence of redundant TE consensus sequences, such as sequences on opposite DNA strands being considered as separate TEs, or multiple consensus sequences being formed from older fragments of a recognizable TE, which are not recognized as the same element in RepeatModeler. However, we suggest that clustering should be used with caution. For example, TEs can transpose into each another, creating new transposition-competent chimeric TEs, which may or may not continue to mobilize as a unit. In such cases, clustering could result in the TE library containing a single consensus for the longest of similar consensus sequences. Consequently, the individual independently mobilizing TEs would be annotated as smaller fragments of the large chimeric TE only and not as separate elements. Conversely, if clustering is not performed, the resultant TE library will contain a consensus sequence for the chimeric TE, as well as sequences for the individual constituent TEs. Additionally, clustering will also mask highly similar TE subfamilies to leave a single representative, which could impact estimates of TE divergence making some TEs appear older than they would if separate subfamilies were retained. Therefore, the default behavior is to avoid clustering, but this can be activated at the discretion of the user.
7. If an initial RepeatMasker step was performed, the curated de novo consensus sequences are combined with the known TE library subset used during the initial RepeatMasker step to produce a combined TE library. Otherwise, the de novo library is used.
8. The de novo TE library (or, optionally, the combined library, produced in step 7) is used to annotate TEs in the input genome using RepeatMasker, with sensitive search settings (which takes longer, but improves TE detection) and ignoring small RNA genes “-s -norna.”
9. The input genome is analysed with LTR_Finder (version 1.07; Xu and Wang 2007), using the LTR_Finder parallel wrapper (Ou and Jiang 2019), to identify full-length LTR elements, which is required for the TE annotation defragmentation performed in step 10.
10. The TE annotations from the final RepeatMasker run are defragmented and combined with the LTR_Finder results using the loose merge “-loose” process in RepeatCraft (Wong and Simakov 2018; https://github.com/niccw/repeatcraftp), which produces a modified GFF file containing the refined TE annotations. Briefly, RepeatCraft identifies nonoverlapping TE annotations from the same TE family in the same orientation (strand) within 150 bp of each other. These annotations are labeled into “groups,” which are then merged to produce a new GFF file with defragmented repeat loci. This improves TE divergence estimates, as remnants of a degraded TE are counted as one insertion rather than several highly degraded fragments, while also reducing inflation of TE copy number.
11. Following defragmentation, Earl Grey removes any overlapping TE annotations using a custom R script (filteringOverlappingRepeats.R, found in the scripts subdirectory of EarlGrey), which assigns half of the length of overlapping segments to each of the two implicated TEs. For example, in the case of a 10 bp overlap between two elements, 5 bp will be assigned to the first TE, and 5 bp will be assigned to the second, thereby ensuring each TE is only annotated and attributed to a single TE.
12. Finally, to decrease the incidence of spurious hits unlikely to be true TE sequences, all TE annotations less than 100 bp in length can be removed before the final set of annotated TEs are quantified. This is at the discretion of the user and can be activated with the flag “-m yes.”
13. Summary figures are generated. Earl Grey automatically produces the following summary figures providing a general overview of TEs in the input genome (Fig. 2): (i) a pie chart illustrating the proportion of the genome assembly annotated with the main TE classifications and non-TE sequence and (ii) a repeat landscape plot, which illustrates the genetic distance between each identified TE and its respective consensus sequence (calculated using the “calcDivergenceFromAlign.pl” utility of RepeatMasker) and is broadly indicative of patterns of TE activity (i.e. recently active TE copies are assumed to have low levels of genetic distance to their respective family consensus). Consistent color keys are used for the pie chart and repeat landscape to facilitate comparison. At the discretion of the user, a softmasked version of the input genome is generated, with annotated TE sequence replaced with lowercase letters in the resulting FASTA file.
14. Upon completion, the main results are saved within the summary files directory, which contains (i) TE annotation coordinates in both GFF3 and Bed formats; (ii) the TE library used for the final annotation; (iii) high-level summary tables quantifying the main TE classifications and a family-level summary to show the most abundant TE families; and (iv) summary figures.

## Materials and Methods

To assess the performance of Earl Grey, we compared it to two widely used existing automated methods: (i) Extensive de novo TE Annotator (EDTA; Ou et al. 2019) and (ii) RepeatModeler2 (Flynn et al. 2020) for de novo TE consensus generation, followed by RepeatMasker (Smit et al. 2013) for genome assembly annotation. To benchmark Earl Grey against these software, we simulated nine genome assemblies where the coordinates and divergence of all TE copies were known, using scripts from Rodriguez and Makałowski (2022) (https://github.com/IOB-Muenster/denovoTE-eval). Briefly, this tool is supplied with a configuration file that specifies genomic GC content, TE sequences to be added, TE copy number, expected TE divergence, percentage of TE copies to be fragmented, and percentage of copies to be nested. An initial random DNA sequence of the specified GC content and overall length is simulated. Then, the TE sequence names and copy numbers are taken, and random positions in the input genome are assigned to each TE. TE sequences are loaded from a supplied library in FASTA format. These sequences are inserted into the base genome sequence at their assigned positions, taking into account the percentage of copies that should be mutated and fragmented. Subsequently, a second script is run, using the same configuration file, to insert nested TEs into existing TEs added to the base genome in the first round, thereby generating nested insertions. A GFF file is produced containing the coordinates of each TE insertion, the divergence of the copy to the original, whether the copy was fragmented, and whether the copy is nested or not. Configuration files used in this study are supplied in supplementary additional file S2.

**Fig. 3.**
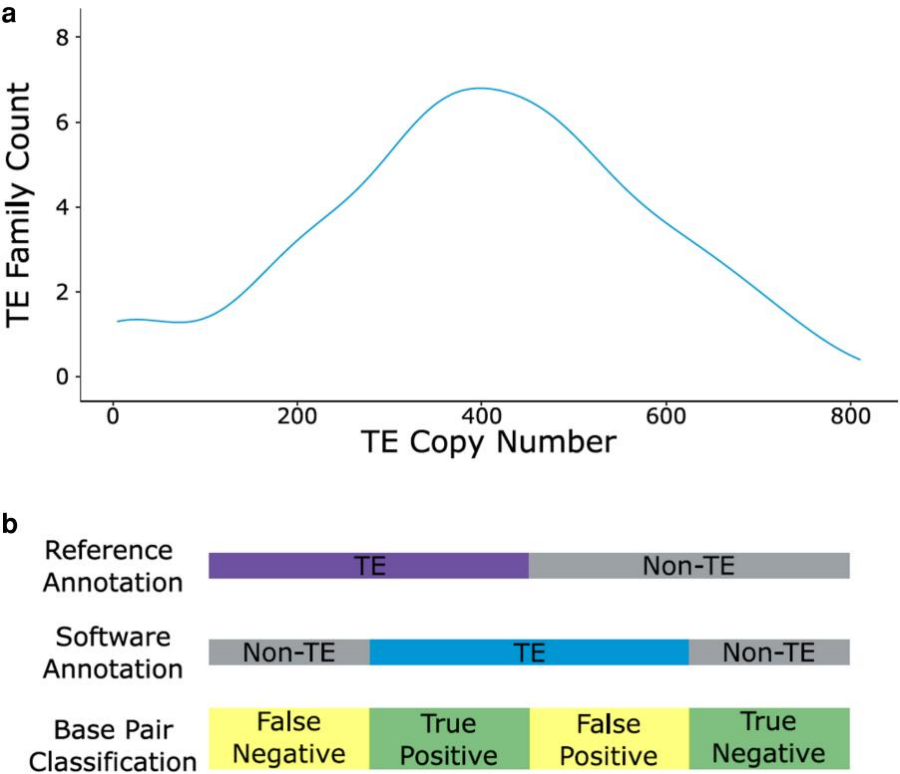
a) Distribution showing the number of TE families across copy numbers in the simulated genomes. b) Schematic indicating the classification of nucleotides annotated by each method in comparison to their true identity.

We generated the nine simulated genomes in a framework that facilitated consideration of the impacts of both total genome size and extremes of GC content. Genomes were generated with initial sizes (before artificial TE insertion) of 250, 400, and 800 Mb, with GC content of 21%, 42%, or 58% (supplementary additional file S2). Low and high GC contents were chosen to reflect extremes observed in real genomes. For example, bacterial GC content can vary from <25% to <75% (Hershberg 2016). Fungi also display large variability in GC content, with the rumen fungus *Neocallimastix californiae* having a very low GC content (18.2%; Peng et al. 2021), while the endophytic fungus *Falciphora oryzae* has a high GC content (56.5%; Xu et al. 2014). Considering sequenced avian, mammalian, and reptilian genomes, GC content tends to lie between 40% and 50% (Bohlin and Pettersson 2019). Therefore, we selected three levels of GC content to reflect TE annotation for the genomes of species from across the tree of life. We inserted 11,883 TE sequences from 30 TE families sourced from Dfam (v3.7; Hubley et al. 2016; Storer et al. 2021) into each of the simulated genome assemblies, including cut, nested, and diverged copies (up to 30% divergence from consensus). Representatives from a variety of TE classifications were selected, including nonautonomous elements such as MITEs (full configuration data sets for each genome are provided in supplementary additional file S2). TE copy number was determined by generating random numbers following a normal distribution, with the lowest TE copy number being 5 and the highest being 732. This generated a distribution close to what we might expect in a “real” genome assembly, where we anticipate that few TE families would be found at low copy number, the majority at intermediate copy number, and few at very high copy number (Fig. 3a). For each simulated genome, the copy number, divergence percentage, nesting percentage, and cutting percentage of each TE family were randomized.

The simulated genomes were annotated with EDTA, RepeatModeler2, and Earl Grey using the default options for all tools. For EDTA, the species flag was set to “others,” as defined in the documentation for analyses of species other than rice or maize. Using scripts developed and described in Rodriguez and Makałowski (2022), TE annotation results for each methodology (“test annotations”) were compared to the “reference annotation” coordinates, detailing the exact position and identity of each TE sequence in the simulated genome, to create a confusion matrix from which the Matthew’s correlation coefficient (MCC) was calculated. An MCC score of +1 arises if all annotations are correct, a score of 0 suggests that test annotations are no better than random guesses, and a score of −1 indicates that all annotations are wrong. Nucleotides were classified based on their agreement between the reference and test annotations as follows: nucleotides found in both reference and test annotations were designated “true positive” (TP), nucleotides absent in both annotations were designated “true negative” (TN), nucleotides found only in the test annotation were designated “false positive” (FP), and nucleotides found only in the reference annotation were designated “false negative” (FN; Fig. 3b; Rodriguez and Makałowski 2022). TE classifications were compared between the reference and the test annotations using BEDTools intersect and subsequent analyses in R using Rstudio and the tidyverse and ape packages (Paradis et al. 2006; Racine 2012; R Core Team 2013; Wickham et al. 2019). To compare each software-generated TE consensus (query) length with the corresponding real TE consensus (subject) length, the highest-scoring pair for each consensus and real sequence was extracted by filtering for the highest bitscore with BLASTn under default parameters (Camacho et al. 2009). Query and subject total lengths were compared to calculate each consensus’ percentage coverage in comparison to total real TE length.

Following initial benchmarking, the genome assembly of the fruit fly, *Drosophila melanogaster* (Release 6.52; Strelets et al. 2014) was annotated to test Earl Grey's performance in a real genomic context.

Overlapping annotations were filtered using a custom R script, and GFF files were compared before and after filtering in R using Rstudio and the tidyverse and ape packages (Paradis et al. 2006; Racine 2012; R Core Team 2013; Wickham et al. 2019). Shared and unique TE annotations were identified using BEDTools intersect (-wao; Quinlan and Hall 2010).

## Results

### Simulated Data Sets: Identification of TEs

The performance of Earl Grey was compared to the following widely used TE annotation methods: (i) EDTA (Ou et al. 2019) and (ii) RepeatModeler2 (Flynn et al. 2020) for de novo TE consensus generation followed by RepeatMasker (Smit et al. 2013) for genome assembly annotation. To assess the relative performance of Earl Grey, we generated nine simulated genomes of varying sizes and GC contents, containing TE insertions extracted from Dfam (see Materials and Methods). The annotations generated by each pipeline were compared to the real coordinates of each TE insertion in the simulated “reference” genome to create confusion matrices from which the MCC was calculated. A score between +1 and −1 is calculated, where +1 indicates a perfect annotation, −1 indicates a totally wrong annotation, and 0 indicates that the annotation is as good as a random guess. Raw annotation files are provided in supplementary additional file S3.

**Fig. 4.**
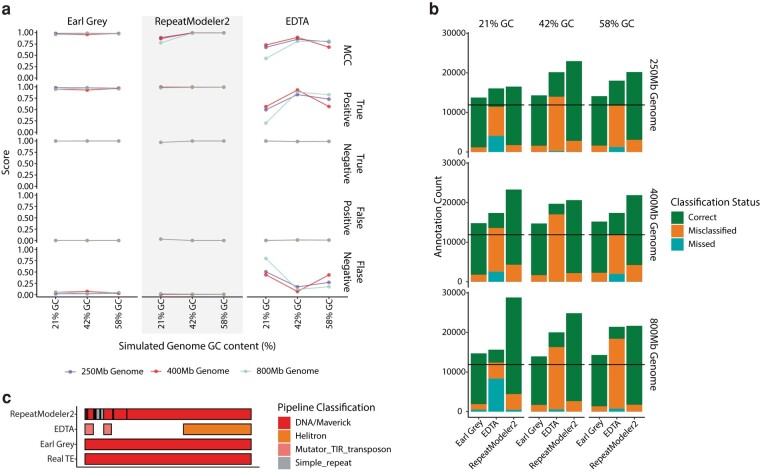
a) MCC and nucleotide classification rates for each method compared using the simulated genomes. Traces show genome size as indicated in the key. b) Number of TE annotations identified by each method with each annotation labeled as classified correctly, misclassified, or missing when compared to the reference TE annotations. Colors in the key indicate classification status for each TE. The black line indicates the real number of TE insertions. c) Schematic demonstrating how the number of TEs annotated can be higher than the real number of TE insertions, as a single TE might be annotated as multiple separate fragments.

In the simulated genomes, Earl Grey and RepeatModeler2 outperform EDTA, with MCC scores averaging 0.97 and 0.94, respectively (Fig. 4). EDTA scored the lowest with an average MCC of 0.67 and has the highest rates of FP and FN annotations (supplementary table S1 and additional file S4). Comparing Earl Grey and RepeatModeler2, the difference in average MCC scores comes from Earl Grey's robust TE annotation in GC-poor (or conversely AT-rich) genomes, where Earl Grey averaged an MCC score of 0.98, while RepeatModeler2 averaged 0.84. Considering mid to high GC content, Earl Grey and RepeatModeler2 both score highly (Fig. 4; supplementary table S1 and additional file S4).

Importantly, MCC scores do not consider whether the annotated TE nucleotides were classified correctly. Rather than just identifying that nucleotides are derived from TE sequence as opposed to host genome sequence, it is also important that TEs are correctly classified as the correct TE type. Therefore, we quantified the number of correct, misclassified, and missing TE annotations for each method. Earl Grey had the highest average correct classification (88.5%) while RepeatModeler2 averaged 86.6% (Fig. 4; supplementary table S2 and additional file S4). EDTA struggled to correctly classify TEs, with a successful classification average of just 23.9% (Fig. 4b; supplementary table S2 and additional file S4). Annotations were much more fragmented when using RepeatModeler2, demonstrated by elevated TE counts compared to the number of actual TE insertions, which arises through a single TE being annotated as multiple separate fragments (Fig. 4b and c).

Overall, Earl Grey performs very well when annotating TEs, with very low FP and FN rates and annotations that closely match the real TE loci, leading to very high MCC scores and high correct classification rates. When assessing the small number of TE insertions missed by Earl Grey (an average of 0.47% of real annotations), we find no systematic bias in their classification.

### Simulated Data Sets: TE Consensus Libraries

Given Earl Grey's high performance in correctly annotating TEs, we next examined how Earl Grey's TE consensus libraries compare to other software that generate de novo TE consensus sequences (namely EDTA and RepeatModeler2).

Thirty real TE families were inserted into the nine simulated genomes. Earl Grey and RepeatModeler2 performed comparably, generating an average of 57.8 and 57.6 consensus sequences, respectively. Earl Grey generated between 49 and 64 consensus sequences, while RepeatModeler2 generated between 47 and 64. EDTA generated the highest number of consensus sequences, with a greatly inflated average of 1,303. EDTA also showed a large range in TE consensus generation, identifying between 21 and 2,948 distinct TE consensus sequences (supplementary table S3 and additional file S4). On average, EDTA identified 796 DNA elements, 169 rolling circle elements, and 338 LTR elements per simulated genome. EDTA did not detect any long interspersed elements (LINEs), short interspersed element (SINEs), or *Penelope-*like elements. Given the high number of consensus sequences generated by EDTA, considering only 30 real TE families were inserted into the simulated genome, we combined the EDTA TE libraries and annotated their consensus sequences with RepeatMasker to interrogate these further. Of the initial 11,727 consensus sequences, 4,903 were annotated with homology to known TEs from Dfam (curated version 3.7) and RepBase (release 20181026). Of these, 3,333 were classified correctly (3,036 LTR, 274 DNA, and 23 rolling circle elements), while 1,570 were misclassified, including several that should be classified as non-LTR retroelements (supplementary table S4 and additional file S4). The remaining 6,824 TE consensus sequences generated by EDTA share no similarity to known TEs in Dfam and RepBase, and so we cannot confirm that they represent real TEs. The exclusion of tools to identify non-LTR retroelements is acknowledged in the original EDTA paper: “Particularly, there is no structure-based program available for the identification of LINEs. The EDTA package may therefore miss a number of elements in, for instance, vertebrate genomes that contain many SINEs and LINEs.” The authors suggest the use of RepeatModeler following EDTA annotation. However, this suggestion is not repeated in the current GitHub repository (https://github.com/oushujun/EDTA) or software documentation and may result in researchers missing this suggestion and assuming that EDTA is a complete TE annotation pipeline suitable for analysing diverse genome assemblies.

**Fig. 5.**
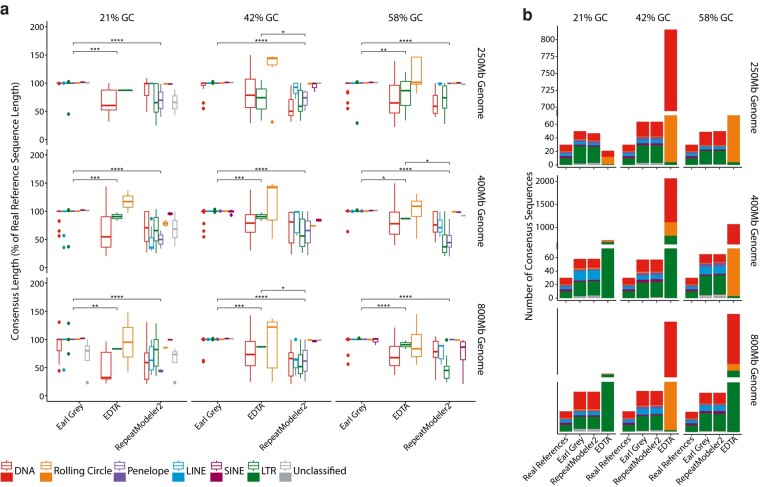
a) TE consensus sequence lengths expressed as a percentage of real TE length for each of the main TE classifications. TE classifications determined by each respective methodology are indicated in the key. Asterisks indicate levels of significance for comparisons of TE consensus length using Wilcoxon rank sum tests (*P≤0.05; **P≤0.01; ***P≤0.001; ****P≤0.0001). Only significant comparisons are shown. For readability, *y* axis limits were restricted to 200% of real sequence length, removing some data points for EDTA. An extended figure can be found in supplementary additional file S6, Supplementary Material online. The DNA TE classification includes DDE-containing TEs, Mavericks/Polintons, and nonautonomous MITEs, as these are labeled as DNA TEs using the RepeatMasker family naming convention (e.g. DNA/Maverick and DNA/Tc1-Mariner will both appear in the DNA category). b) Number of consensus sequences generated by each methodology, along with the number of real reference sequences. EDTA consensus sequence counts required axis-scale adjustment due to the very high consensus sequence number generated. Colors show major TE classifications, as indicated in the key.

A key aim of Earl Grey is to generate longer consensus sequences, but without extending them significantly beyond real TE boundaries. In all simulated genomes, Earl Grey does not generate TE consensus sequences that are significantly different in length from the real reference TE sequences (supplementary table S5 and additional files S4 and S5). Further, when considering all TE consensus sequences, Earl Grey generates significantly longer consensus than both RepeatModeler2 and EDTA (supplementary table S5 and additional files S4 and S5), suggesting that these pipelines underestimate TE consensus length and thus can miss considerable proportions of true TE sequence within a genome. This is further supported when comparing the consensus sequences generated by each method against the reference TE sequences. Specifically, 88.50% (454/513) of the total consensus sequences generated by Earl Grey are between 95% and 105% of the real TE sequence length, while only 21.72% (111/511) of RepeatModeler2 consensus sequences and just 4.49% (23/512) of EDTA consensus sequences are within this length threshold (Fig. 5a; supplementary table S6 and additional file S4). Generally, RepeatModeler2 generates TE sequences below the real TE length, with 77.30% (395/511) of RepeatModeler2 consensus sequences below 95% of real TE consensus length. Conversely, EDTA consensus sequences vary considerably in length compared to real consensus sequences, with EDTA generating sequences between 20% and 7,040% of the length of their corresponding real TE sequences, with 68.36% (350/512) being over 105% of the real TE consensus length and 27.15% (139/512) being <95% of the real TE consensus length (Fig. 5a; supplementary table S6 and additional file S4). Earl Grey therefore represents an improvement over RepeatModeler2 and EDTA in approaching full-length TE consensus sequences without significant overextension past real TE boundaries. Considering individual classifications, Earl Grey generates significantly longer LTR consensus sequences than RepeatModeler2 irrespective of genome size or TE content while generating significantly longer LINE (400 Mb 21% and 42% GC; 800 Mb 42% GC) and DNA consensus sequences (250 Mb 42% GC; 800 Mb 42% GC) in certain genomic contexts (Fig. 5a; supplementary table S5 and additional file S4). LTR consensus sequences generated by RepeatModeler2 were significantly shorter than the real reference sequences in all simulated genome assemblies except for the 800 Mb genome with 21% GC content, in which the LINE consensus sequences were significantly shorter (Fig. 5a; supplementary table S5 and additional file S4). When compared to EDTA, we find that Earl Grey generates significantly longer LTR consensus sequences (400 Mb 21% and 42% GC; 800 Mb 21% and 58% GC; Fig. 5a; supplementary table S5 and additional file S4). EDTA LTR consensus sequences were significantly shorter than the real reference sequences in larger simulated genomes (400 Mb 21% and 42% GC; 800 Mb 21% and 58% GC). EDTA generated significantly longer DNA consensus sequences than RepeatModeler2 in the 800 Mb simulated genome with 42% GC content (Fig. 5a; supplementary table S5 and additional file S4). However, this result should be taken with caution given the high levels of misclassification observed with EDTA. The generation of longer consensus sequences in Earl Grey can be attributed to the automated implementation of the iterative BEAT process that seeks to generate maximum-length consensus sequences from the initial de novo TE consensus sequences identified by RepeatModeler2 while ensuring consensus sequences encompass true TE boundaries and minimize the inclusion of sequence beyond these.

### Simulated Data Sets: TE Annotations

**Fig. 6.**
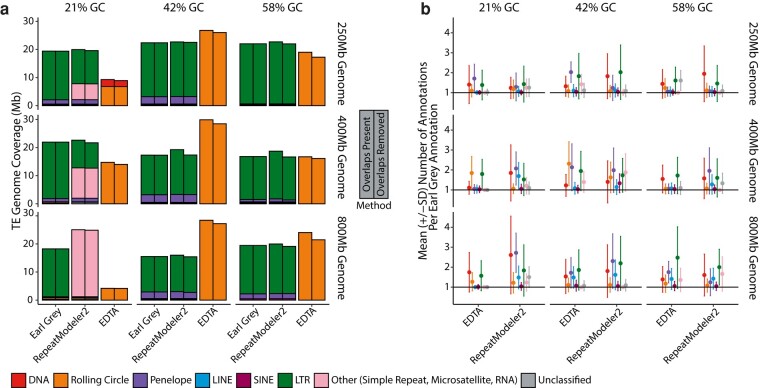
a) Number of TEs annotated in the simulated genomes before and after overlapping annotations have been removed. For each method, left-hand bars show TE coverage including overlapping annotations, while right-hand bars indicate TE coverage following removal of overlaps. TE classifications are indicated in the key. b) Number of annotations per Earl Grey annotation for each simulated genome, demonstrating the level of fragmentation in TE annotation. The black line at 1 indicates the number of annotations expected if annotations are no more fragmented than Earl Grey. TE classifications, as identified by Earl Grey, are indicated in the key.

As discussed above, improving the accuracy of TE consensus sequence can have a considerable impact on consensus length and consequently the amount of correctly annotated TE sequence within a genome. Additionally, Earl Grey was developed to improve several other aspects of TE annotation. Firstly, Earl Grey aims to address current issues with overlapping TE annotations. It is important to address overlapping annotations to prevent inflation of TE count and coverage estimates, as it is not physically possible for a single locus to belong to multiple TEs. When interrogating final annotation outputs, we find overlapping annotations in the outputs of EDTA and RepeatMasker (used to annotate TEs generated with RepeatModeler2), which inflate TE count and coverage estimates. The greatest amount of overlapping annotated TE sequence was found when using EDTA, where removing overlaps reduced overall TE coverage by an average of 7.8% (ranging from 2.4% to 12.7%; Fig. 6a; supplementary table S7 and additional file S4) and TE count by an average of 4.8% (ranging from 1.9% to 8.3%; supplementary table S7 and additional file S4).

Secondly, Earl Grey aims to address current issues with the fragmentation of TE annotations, where a single TE can be annotated as multiple fragments, for example due to degradation of the TE sequence. This can inflate estimates of TE copy number, and it can also complicate investigations into TE association with host gene features. To address this, Earl Grey includes a postannotation process employing RepeatCraft (Wong and Simakov 2018) to merge annotations that are likely to belong to the same TE insertion. When examining annotation fragmentation, we find that all methods produce annotations that are more fragmented than Earl Grey, indicated by a mean number of annotations per Earl Grey annotation of more than one for all TE classifications (Fig. 6b). Levels of fragmentation were comparable between EDTA and RepeatMasker (using the RepeatModeler2 consensus library), with an average of 1.33 and 1.42 annotations per Earl Grey annotation, respectively (Fig. 6b).

### A Test Using a Real Genome Assembly: TE Consensus Library Quality

Following benchmarking of Earl Grey using simulated genomes, we annotated the genome of *D. melanogaster* (Release 6.52; Strelets et al. 2014) to compare Earl Grey to other software in a real genome assembly. TE annotations from each pipeline are provided in supplementary additional file S7.

**Fig. 7.**
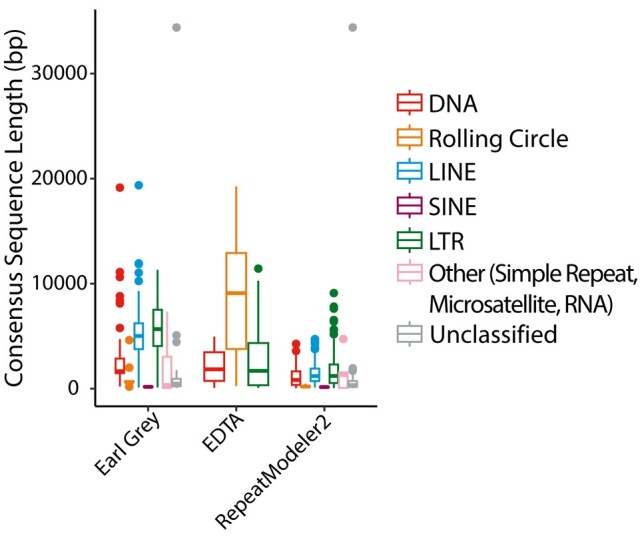
Length of TE consensus sequences split by TE classification and consensus generation methodology. EDTA results should be taken with caution given widespread instances of TE misclassification identified in the simulated genomes. TE classifications are indicated in the key in box order and colour.

**Fig. 8.**
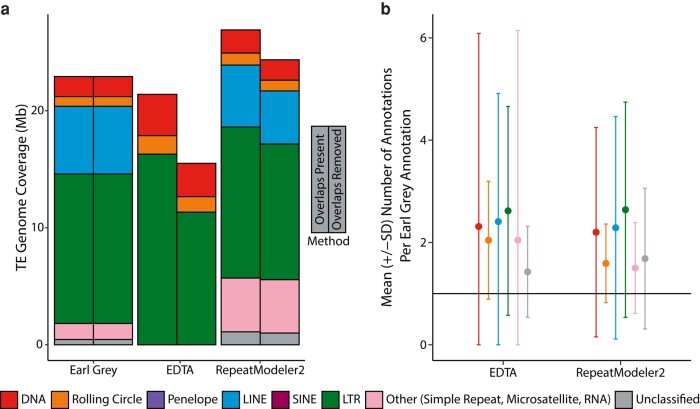
TE classifications are indicated by the color in the key. a) TE coverage expressed as percentage of the total *D. melanogaster* genome assembly annotated using each methodology. Coverage before and after removing overlapping annotations is shown, with TE types colored as indicated in the key. b) Mean number of TE annotations per Earl Grey annotation at shared loci. Error bars indicate 1 SD from the mean (circles). The black line indicates a perfect match where one annotation matches one annotation using Earl Grey.

Earl Grey and RepeatModeler2 each generated 388 consensus sequences. However, there were fewer unclassified consensus sequences when using Earl Grey (Earl Grey: 88% classified; RepeatModeler2: 81% classified; supplementary table S8 and additional file S4). Meanwhile, EDTA generated 583 consensus sequences (supplementary table S8 and additional file S4; Fig. 7). With the exception of rolling circle elements, SINEs, and unclassified elements, Earl Grey also generated significantly longer TE consensus sequences (DNA: Kruskal–Wallis, *χ*^2^_2_ = 21.3, *P* < 0.01; LTR: Kruskal–Wallis, *χ*^2^_2_ = 204.0, *P* < 0.01; LINE: Wilcoxon rank sum, *W* = 5578.5, *P* < 0.01; unclassified: Wilcoxon rank sum, *W* = 1919.5, *P* < 0.01; Fig. 8). This can be attributed to the automated implementation of the iterative “BEAT” process in Earl Grey that works to generate maximum-length consensus sequences from initial de novo TE consensus sequences identified by RepeatModeler2. The significantly longer rolling circle consensus sequences generated by EDTA (Kruskal–Wallis, *χ*^2^_2_ = 48.1, *P* < 0.01) should be treated with caution due to the very high levels of misclassification observed when using EDTA in the simulated genomes, with these consensus sequences likely instead belonging to a variety of different TE classifications. No significant difference in consensus length between Earl Grey and RepeatModeler2 was found for SINEs and unclassified elements (SINEs: Wilcoxon rank sum, *W* = 2.5, *P* = 1; unclassified: Wilcoxon rank sum, *W* = 1919.5, *P* = 0.15).

### A Test Using a Real Genome Assembly: TE Annotation Quality

When comparing TE annotation results, we find higher TE proportions annotated when using Earl Grey in comparison to EDTA (Fig. 8; supplementary table S9 and additional file S4). However, RepeatMasker used with the RepeatModeler2 de novo TE library led to higher proportions of the genome being annotated as TE than Earl Grey in *D. melanogaster* (Fig. 8; supplementary table S9 and additional file S4).

The highest abundance of unclassified elements was annotated when using RepeatModeler2. This is due to RepeatModeler2 annotating putative TE sequences that do not share sufficient similarity with the TEs in Dfam and RepBase to enable their classification using the RepeatClassifier module. This is surprising given the abundance of knowledge regarding the TE content of *D. melanogaster*, which has been a focus of TE research for at least 43 yr (Green 1980; Mérel et al. 2020) and for which substantial TE resources exist. Earl Grey also makes use of the RepeatClassifier module, but it does so with longer and more refined consensus sequences, which likely reveals sufficient homology to known elements to enable their classification.

When interrogating final annotation outputs, we find numerous overlapping annotations in the outputs of EDTA and RepeatMasker (using the RepeatModeler2 library), which inflate TE count and coverage estimates (Fig. 8; supplementary table S9 and additional file S4). For EDTA, removing these overlaps reduces total annotation coverage by 22%, while annotation coverage is reduced by 9.5% for RepeatMasker (using the RepeatModeler2 library; Fig. 8).

Annotations produced with RepeatMasker (using the RepeatModeler2 library) and EDTA are more fragmented than Earl Grey annotations. This is demonstrated by each Earl Grey annotation, of those that are shared, overlapping with multiple annotation loci generated by the other methodologies (Fig. 8). Specifically, each Earl Grey annotation was represented by an average of 2.14 annotations when using EDTA and 1.98 annotations when using RepeatMasker (with the RepeatModeler2 library). For all TE classifications, the level of fragmentation was reduced when using Earl Grey, demonstrating Earl Grey's ability to defragment repeat annotations in a real genomic context.

## Discussion

We have introduced Earl Grey, a fully automated TE annotation and analysis pipeline for repeat identification in genome assemblies of diverse organisms. Earl Grey provides various benefits over other pipelines employed for TE annotation. Specifically, Earl Grey was designed to increase TE consensus sequence length, resolve spurious overlapping and fragmented annotations, offer user-directed flexibility in TE annotation parameters, provide users with results in standard formats for compatibility with downstream analyses, and improve the user-friendliness of TE annotation. In addition, “paper-ready” summary figures are produced to provide researchers with a high-level overview of the TE landscape and activity profile for a given genome assembly.

Benchmarking of Earl Grey shows favorable improvements in TE annotation compared to other commonly used pipelines, with an MCC very close to a perfect score of +1. In addition to being robust in terms of TE consensus generation and subsequent TE annotation, Earl Grey will benefit researchers requiring TE annotation in an “all-in-one” automated package requiring no extra analysis tools or steps. Although not as accurate as Earl Grey, RepeatModeler2 scored favorably in benchmarking, but it requires a separate RepeatMasker run following TE library generation to annotate a genome. In addition, Earl Grey is more robust than existing methods when analyzing AT-rich genomes, making it suitable for annotation of genome assemblies representing diverse species from across the eukaryotic tree of life.

Earl Grey provides extra polishing steps to further optimize TE annotation results. Through the implementation of the automated “BEAT” process, Earl Grey succeeds in producing longer TE consensus sequences without extending significantly beyond true element boundaries (Fig. 7b). In the context of TE annotation, the longer TE consensus sequences generated by Earl Grey are beneficial in comparison to the shorter ones produced by the other software, as finding longer matches between individual putative TE copies in a genome brings us closer to confident identification of TE boundaries and more accurate estimations of genome-wide TE content, which remains a significant challenge within the TE field and is of direct utility for various research applications. There is little chance of the TE boundary sequence being annotated if it is not found in the shorter TE consensus sequences generated by other software. Therefore, finding longer matches among individual putative TE copies in a genome, without extending to generate consensus sequences longer than real insertions, presents a step forward in our ability to accurately identify TEs in genome assemblies. In some instances, some TE consensus sequences can still be slightly overextended through the automated process; therefore, care should still be used when further curating specific TE families of particular interest in a given study. It should be noted that we assessed the performance of RepeatModeler2 under default settings. There is an optional LTR module available for RepeatModeler2, which is likely to improve the performance of RepeatModeler2 for LTR consensus generation in terms of LTR sequence length and completeness.

As previously discussed by Rodriguez and Makałowski (2022), redundancy in the models generated by existing de novo TE identification tools is an issue during annotation. We have addressed this in Earl Grey through the inclusion of optional library redundancy reduction steps. By clustering after the BEAT process, only TE sequences from different putative TE families are included in the final TE consensus library. However, this remains an optional step in the Earl Grey pipeline and should be used with caution, including manual evaluation of redundancies.

When considering the extent of overlap in annotations produced by other methods, Earl Grey demonstrates a significant improvement over existing pipelines by eliminating overlapping TE annotations. This reduces the risk of inflated TE counts while remaining accurate in estimating TE coverage in genome assemblies. Furthermore, the removal of overlapping annotations ensures that each base pair of the genome assembly is only annotated as a single TE, so that results remain biologically plausible.

## Future Developments

A key aim of Earl Grey was to produce a user-friendly TE annotation pipeline that can facilitate large-scale comparative studies through a fully automated process. This first release of Earl Grey provides an improved starting point for automated TE curation and annotation, which can be built upon, to meet the developing needs of the genomics research community. To this end, we have identified several areas of development to be considered for future implementation.

While efforts have been made to improve current automated curation methodologies, we acknowledge that for the foreseeable future, the gold standard will remain manual curation. However, this is also possible following Earl Grey analysis, which can be used to accelerate the process, as demonstrated previously for an in-depth annotation of the monarch butterfly genome (Baril and Hayward 2022).

Features of the biology of certain TEs can make automatic curation particularly challenging, and several challenges remain. For example, many LINE insertions (and *Penelope*-like element [PLE] insertions) are heavily 5′ truncated due to premature termination of reverse transcription during first-strand synthesis, either as a consequence of polymerase inefficiency or host cleavage of the RNA intermediate (Suzuki et al. 2009; Baldwin et al. 2024). In situations where there is an accumulation of 5′ truncated elements and the retention of only very few full-length sequences, automated curation can result in a 5′-incomplete LINE consensus sequence, given insufficient numbers of columns with identities in the 5′ regions of the MSAs used to compute the consensus (Fig. 5a). To address this, future improvements could involve the differential treatment of the 5′ and 3′ ends of putative LINE consensus sequences to allow extension of the 5′ end with fewer column identities in the alignment compared to the 3′ end.

There are inherent limitations with all-by-all genome alignment methods for the identification of repetitive elements. In particular, very low copy number TE families evade detection due to the thresholds used to automatically define “repetitive sequences.” If resources are available, some low copy number TE families can be detected using a population or pangenomic approach, which can enable all-by-all comparison algorithms to detect these in several copies, as used to generate a comprehensive TE library for the fungal pathogen, *Zymoseptoria tritici* (Baril and Croll 2023).

Currently, Earl Grey assigns 50% of overlapping base pairs to each of the overlapping TE annotations to resolve overlaps. In an optimal scenario, it would be beneficial to determine which TE each overlapping base belongs to. While this seems like a simple issue to solve on the surface, it actually represents a deeply complex problem that is difficult to solve with existing approaches. Overlaps arise when the bases match with similar scores to competing annotations, meaning that existing algorithms cannot determine which base belongs to which TE. To overcome this, novel approaches are needed that can resolve similar-scoring base pairs to the correct TE. In the short term, approaches using windowed alignment scores, or score density, can help to resolve such cases. Further, alignment comparison approaches such as PolyA (Carey et al. 2021) could also aid in correct TE boundary resolution among conflicting loci. Future approaches may involve consideration of interclassification differences in TE dynamics and degradation patterns to make informed predictions on the likelihood of bases belonging to one TE rather than another. However, this will require significant improvements in our understanding of TE–host dynamics and a movement away from BLAST-based annotation to approach this problem.

We retain the RepeatMasker TE consensus naming convention in Earl Grey to maximize compatibility with other commonly used tools. However, we acknowledge the shortfalls of this system, which have become apparent as understanding of TE evolution and diversity has developed. Consequently, users should be aware of the limitations associated with the current naming convention, and we recommend that they should always consult the full TE classification names when considering TE type. For example, the RepeatMasker “DNA” classification includes DDE-containing DNA TEs, nonautonomous DDE DNA TEs, their much smaller derivative nonautonomous MITEs, and the much larger and evolutionarily divergent Maverick/Polinton TEs. Further, *Penelope*-like elements are designated as “LINE/Penelope,” despite their different transposition mechanisms.

A major challenge in identifying de novo TEs in genome assemblies concerns the annotation of non-TE sequences. Many de novo methods, including those employed by Earl Grey, work by identifying sequences found in multiple copies in the genome. As TE annotation is often performed prior to gene annotation, care should be taken that multicopy genes are not incorrectly designated as TE sequences. For example, this can occur when annotating TEs in animals, where olfactory receptor genes are frequently duplicated and exist in expanded copy number families (Mombaerts 1999). To overcome this, gene annotations or models can be used to retain genomic loci known to contain these genes of interest prior to TE annotation, although this can only be done if such models exist, and prior knowledge is held. To address this, we plan to develop a module that will identify multicopy genes and prevent them ending up in TE consensus libraries.

The advancement of genome sequencing technology has been accompanied by a corresponding decrease in sequencing costs. Consequently, the vast majority of genome assemblies being released today are chromosome level. For example, the Darwin Tree of Life project aims to release ∼70,000 high-quality de novo chromosome-level genome assemblies (https://www.darwintreeoflife.org/). With the release of chromosome-level assemblies comes distinct opportunities to further characterize TE landscapes. To add to the current summary plots generated by Earl Grey, it would be informative to automatically generate karyoplots to show the chromosomal distribution of the main TE classifications across the genome assembly (e.g. see examples in Li et al. 2020; Baril and Hayward 2022). As well as providing a visual aid, these can help to identify areas of interest for further interrogation. In addition to visual additions, there are opportunities to present the chromosomal distribution of TEs within a quantitative framework. An additional module for Earl could identify statistically significant hotspots and coldspots for TE insertion, where TEs are found in higher, or lower, densities than expected if TEs were evenly distributed across the host genome (Baril and Hayward 2022). To build on this and enable comparisons across higher taxonomic levels, a metric for “evenness of spread” can be included (Baril and Hayward 2022). This can assess the distribution of TEs within a given context and provide an estimate of how even the observed distribution of TE sequences is in a cross-comparable manner.

Here, we have presented Earl Grey, a new fully automated TE annotation pipeline for the annotation of genome assemblies. We have shown that Earl Grey improves upon current widely used TE annotation methodologies in terms of consensus generation, removing overlapping annotations, and reducing fragmentation of annotations. Earl Grey will help to facilitate large-scale comparative genomic studies, as well as being user-friendly and producing outputs in common formats compatible with downstream analyses. There remains scope for further improvements of Earl Grey, especially via incorporating suggestions and feedback from the needs of the research community. Finally, Earl Grey is an open-source project hosted on GitHub. Our aim is for the TE community to request and contribute new features and improvements to the Earl Grey project so that it is a community-led effort to improve TE annotation.

## Availability and Requirements

Project name: Earl Grey.

Project home page: https://github.com/TobyBaril/EarlGrey.

Operating systems: Linux-based systems (e.g. Ubuntu), MacOS-based systems (OSX), or a web browser (for the gitpod implementation).

Programming language: Pipeline including software coded in Python, R, Bash, and Perl.

Other requirements: Anaconda3, Docker (optional), and Singularity (optional).

License: Open Software License v 2.1

Restrictions for nonacademic users: none. Some dependencies may require licenses for use by nonacademic users.

## Supplementary Material

msae068_Supplementary_Data

## Data Availability

All data generated or analyzed during this study are included in this published article and its supplementary information files, which are hosted on Zenodo under the DOI:10.5281/zenodo.10683832 (https://zenodo.org/doi/10.5281/zenodo.10683832).
